# Home working and social and mental wellbeing at different stages of the COVID-19 pandemic in the UK: Evidence from 7 longitudinal population surveys

**DOI:** 10.1371/journal.pmed.1004214

**Published:** 2023-04-27

**Authors:** Jacques Wels, Bożena Wielgoszewska, Bettina Moltrecht, Charlotte Booth, Michael J. Green, Olivia KL Hamilton, Evangelia Demou, Giorgio Di Gessa, Charlotte Huggins, Jingmin Zhu, Gillian Santorelli, Richard J. Silverwood, Daniel Kopasker, Richard J. Shaw, Alun Hughes, Praveetha Patalay, Claire Steves, Nishi Chaturvedi, David J. Porteous, Rebecca Rhead, Srinivasa Vittal Katikireddi, George B. Ploubidis

**Affiliations:** 1 MRC Unit for Lifelong Health and Ageing, University College London, London, United Kingdom; 2 Centre Metices, Université libre de Bruxelles, Brussels, Belgium; 3 Centre for Longitudinal Studies (CLS), Social Research Institute, University College London, London, United Kingdom; 4 MRC/CSO Social and Public Health Sciences Unit, School of Health and Wellbeing, University of Glasgow, Clarice Pears Building, Glasgow, United Kingdom; 5 Research Department of Epidemiology and Public Health, University College London, London, United Kingdom; 6 Centre for Genomic and Experimental Medicine, The University of Edinburgh Western General Hospital, Edinburgh, United Kingdom; 7 Bradford Teaching Hospitals NHS Foundation Trust, Bradford, United Kingdom; 8 Twin Research & Genetic Epidemiology, King’s College London, St Thomas’ Hospital London, United Kingdom; N/A, UNITED KINGDOM

## Abstract

**Background:**

Home working has increased since the Coronavirus Disease 2019 (COVID-19) pandemic’s onset with concerns that it may have adverse health implications. We assessed the association between home working and social and mental wellbeing among the employed population aged 16 to 66 through harmonised analyses of 7 UK longitudinal studies.

**Methods and findings:**

We estimated associations between home working and measures of psychological distress, low life satisfaction, poor self-rated health, low social contact, and loneliness across 3 different stages of the pandemic (T1 = April to June 2020 –first lockdown, T2 = July to October 2020 –eased restrictions, T3 = November 2020 to March 2021 –second lockdown) using modified Poisson regression and meta-analyses to pool results across studies. We successively adjusted the model for sociodemographic characteristics (e.g., age, sex), job characteristics (e.g., sector of activity, pre-pandemic home working propensities), and pre-pandemic health. Among respectively 10,367, 11,585, and 12,179 participants at T1, T2, and T3, we found higher rates of home working at T1 and T3 compared with T2, reflecting lockdown periods. Home working was not associated with psychological distress at T1 (RR = 0.92, 95% CI = 0.79 to 1.08) or T2 (RR = 0.99, 95% CI = 0.88 to 1.11), but a detrimental association was found with psychological distress at T3 (RR = 1.17, 95% CI = 1.05 to 1.30). Study limitations include the fact that pre-pandemic home working propensities were derived from external sources, no information was collected on home working dosage and possible reverse association between change in wellbeing and home working likelihood.

**Conclusions:**

No clear evidence of an association between home working and mental wellbeing was found, apart from greater risk of psychological distress during the second lockdown, but differences across subgroups (e.g., by sex or level of education) may exist. Longer term shifts to home working might not have adverse impacts on population wellbeing in the absence of pandemic restrictions but further monitoring of health inequalities is required.

## Abstract 2

Jacques Wels and colleagues investigate the relationship between working from home and different indicators of mental and social wellbeing in the UK.

## Author summary

**Why was this study done?:**

➢ The number of workers working from home drastically increased during the Coronavirus Disease 2019 (COVID-19) pandemic.
➢ Little is known about the relationship between home working and mental and social wellbeing before and during the pandemic.
➢ As some home working schemes are likely to remain post-pandemic, understanding this association is important.

**What did the researchers do and find?:**

➢ We analysed data from 7 UK-based population surveys to better understand the relationship between working from home (partially or fully) and different indicators of mental and social wellbeing.
➢ We analysed these relationships over 3 time points—i.e., April to June 2020 (T1), July to October 2020 (T2), and November 2020 to March 2021 (T3)—and controlled for sociodemographic (e.g., age, sex, and ethnicity) and job characteristics (e.g., sector of activity, key worker status).
➢ We found that home working was not associated with psychological distress at T1 or T2, but at T3 it was associated with increased psychological distress (relative risk = 1.17, 95% CI = 1.05 to 1.30). No other meaningful associations were found.
➢ We stratified these associations by sex, education level, age, and full-time versus part-time employment and found that home working was associated with greater psychological distress in those with lower than degree level education.

**What do these findings mean?:**

➢ We found no clear evidence of a lasting association between home working, mental health, and social wellbeing which may indicate that, during the pandemic, home working was not detrimental nor beneficial for workers’ wellbeing.
➢ Differences across subgroups may exist and specific impacts by sex, age, and education level need to be investigated further.

## Introduction

Home working is rapidly increasing worldwide [1–5]. This shift had largely been voluntary until the Coronavirus Disease 2019 (COVID-19) pandemic, when home working became mandatory for many workers. According to the International Labour Organisation, 557 million employees worldwide worked from home during the second quarter of 2020, accounting for 17 percent of the global workforce [6]. In the United Kingdom, estimates were higher, with 37 percent of the workforce working from home in 2020 compared to the 27 to 30 percent who worked from home in 2019 [7,8]. This sudden and widespread uptake in home working provides an opportunity to examine the potential impact of home working on the mental health and wellbeing of a diverse range of workers. This is of particular importance if higher levels of home working are sustained. Over the period April to May 2022, there were 38% of working adults reporting hybrid working in the UK, even after the working from home guidance was lifted, compared to 12% prior to the pandemic [9].

The relationship between home working and mental health is poorly understood, with mixed pre-pandemic and pandemic evidence and potential mechanisms for both positive [10–13] and negative impacts [14–16]. Potential impacts of home working on health inequalities have been under-explored in the literature, so it is important to assess whether associations differ by social factors such as sex, age, education, and hours worked. The pandemic context also offers the opportunity to assess the relationship between home working and mental health under varying degrees of public restrictions, at different time points during the pandemic.

The UK National Core Studies Longitudinal Health and Wellbeing initiative draws together data from several UK population-based longitudinal studies, using coordinated analyses to answer pandemic-related questions. By conducting new harmonised analyses within each study and pooling estimates, we provide robust evidence on associations between home working and mental wellbeing during the pandemic, with a view to understanding the longer-term consequences of this shift. More specifically, we address the following 2 research questions (RQs): (RQ1) Is working from home (fully or partially) associated with psychological distress, low life satisfaction, low self-rated health, low social contact, and loneliness at different stages of the pandemic?. (RQ2) Do associations between home working and self-reported social and mental wellbeing differ by sex, age, education, or full-time versus part-time work?

## Methods

### Ethics statement

The most recent sweeps of the NCDS, BCS70, and Next Steps have all been granted ethical approval by the National Health Service (NHS) Research Ethics. The University of Essex Ethics Committee has approved all data collection for the Understanding Society main study and COVID-19 waves. Waves 1–9 of ELSA were approved through the National Research Ethics Service, while the COVID-19 Sub-study was approved by the UCL Research Ethics Committee. Generation Scotland obtained ethical approval from the East of Scotland Committee on Medical Research Ethics (on behalf of the NHS). Reference number 20/ES/0021. Ethical approval for the Born in Bradford study was granted by the National Health Service Health Research Authority Yorkshire and the Humber (Bradford Leeds) Research Ethics Committee (reference: 16/YH/0320). For all studies, respondents gave an informed consent. Further details on informed consent are mentioned in S1 Supplementary File.

### Data, design, and sample

We conducted primary data analyses in 7 UK population-based studies, including 3 age-homogenous birth cohorts: Next Steps (NS, formerly the Longitudinal Study of Young People in England), the 1970 British Cohort Study (BCS70), and the 1958 National Child Development Study (NCDS); and 4 age heterogenous studies: Understanding Society—also referred to as the UK Household Longitudinal Study (USOC), the English Longitudinal Study of Ageing (ELSA), the Scottish Family Health Study—Generation Scotland (GS), and Born in Bradford (BiB). Details of all studies as well as ethical approvals are presented in S1 Supplementary File.

Participants were surveyed at multiple time points during 3 key periods (data availability by time point in S2 Supplementary File). The first period (T1) included surveys from April to June 2020, during the initial surge of infections and the first national lockdown. The second period (T2) included surveys from July to October 2020, as initial restrictions were eased. The final period (T3) included surveys from November 2020 to March 2021, as infection rates rose again, and a second national lockdown was introduced.

Analytical samples included respondents of working age (16 to 66) who were employed (i.e., excluding the self-employed) prior to the pandemic and actively employed (i.e., excluding those who were furloughed) during at least one of the pandemic time points. The sample was restricted to complete cases and to respondents for whom information about mental health and social wellbeing was collected in both pandemic and pre-pandemic surveys.

All analyses were preplanned and registered in a published protocol (https://archive.org/details/osf-registrations-49ksd-v1) [17]. This study is reported as per the Strengthening the Reporting of Observational Studies in Epidemiology (STROBE) guideline (S1 STROBE Checklist).

### Measures

Measures and derived harmonised variables are described below, with further details on study-specific measurement in S3 Supplementary File.

#### Exposure: Home working

This study used a self-reported definition of home working that is independent of the form it may take. Respondents in each study indicated whether they had been working from home fully, partially, or not at all, at each of the 3 pandemic time points. As NS, BCS70, and NCDS did not collect information about partial home working at T1, across all studies, we generated a “home working” variable with 2 modalities at T1 (0 = “working entirely at employer’s premises or other location”; 1 = “working fully or partially from home”) and 3 modalities at T2 and T3 (0 = “working entirely at employer’s premise or other location”; 1 = “partially working from home”; 2 = “fully working from home”).

#### Outcomes: Social and mental wellbeing

We investigated 5 different outcomes: psychological distress, low life satisfaction, poor or fair self-rated health, low social contact (including online contacts), and loneliness. For each outcome, we created a binary indicator using pre-validated cut-off scores where possible (detailed information about measurement across studies in S3 Supplementary File).

#### Covariates

Analyses for RQ1 were repeated with 4 nested levels of adjustment, with each including a progressively larger set of covariates variables:

1. No adjustment.
2. Sociodemographic adjustment: age (for age-heterogeneous studies; 3 categories: 16 to 29, 30 to 49, 50+ that maximise population sizes by group), sex (male, female), housing tenure (mortgage or owner versus other), ethnicity (white—including white minorities—versus ethnic minority groups), level of education (university degree versus lower level of education), and household overcrowding (number of people in household/number of rooms). We also added a household composition variable with 6 categories, in line with the evidence suggesting women have been disproportionately burdened with childcare and home schooling during the pandemic [18,19] (“alone” (reference); “1 = female with partner and child(ren)”; “2 = male with partner and child(ren)”; “3 = female with partner and no child(ren)”; “4 = male with and partner no child(ren)”; “5 = lone parent”; “6 = others (i.e., living with other relatives or nonrelatives)”).
3. Job adjustment: additionally included pre-pandemic weekly working hours, pre-pandemic socioeconomic position (3 class National Statistics Socio-Economic Classification; NS-SEC), 2-digit Standard Occupational Classification (SOC), and key worker status during the pandemic. We also controlled for propensity to be working from home prior to the start of the pandemic. Propensities were derived from the Annual Population Survey [20] because no study except USOC collected information on it. These were calculated as the propensities to work fully or partially from home based on SOC2010 (2-digits), sex, and age-group (16 to 29, 30 to 49, and 50 to 66) prior to the start of the pandemic (April 2019 to March 2020), as described in S4 Supplementary File.
4. Full adjustment: additionally controlled for pre-pandemic mental health, social wellbeing, and the presence of a long-standing illness or disability, as described in S3 Supplementary File. The fully adjusted model controls for pre-pandemic measures of the outcome and the resultant association can be interpreted as change in the outcomes compared to pre-pandemic levels.

Analyses for RQ2, which were stratified by sex, age, education level, and part-time versus full-time work used the full adjustment only.

### Analyses

For RQ1, we first ran within-study modified Poisson regression models with robust standard errors to model binary outcome variables [21,22] and report risk ratios (RR). RRs—also called relative risks or relative risk ratios—ease interpretation and avoid issues related to non-collapsibility of odds ratios [23]. USOC had multiple surveys within each time period, so multilevel models were used to account for correlation between responses from the same individuals. We modelled the outcomes at each time point separately, using weights to account for survey nonresponse [24]. Nonresponse weights were derived in each study separately. Variables were included in the response model on the basis of their a priori assumed association with the probability of response (based on the existing literature regarding survey response) and/or with key COVID-19 survey variables. Estimates from each model and study were then pooled using a random effects meta-analysis with restricted maximum likelihood.

Sensitivity analyses were conducted to validate the imputed propensity for pre-pandemic home working using data from USOC where actual reported pre-pandemic home working was available, to check for consistency with the imputed variable. Models for RQ1 were also repeated excluding data from GS and BiB (which did not have sufficient data for all levels of adjustment), to check estimates consistency.

For RQ2, stratified analyses were conducted in the same way as above with subgroup meta-analyses performed by sex (male, female), age group (16 to 29, 30 to 49, 50+), education level (university degree, lower education level), and working hours (full-time, part-time) in order to assess between-group differences in the association between home working and social and mental wellbeing.

Study-specific analyses were conducted using either R or Stata. Meta-analyses were conducted using Stata.

## Results

Across the 7 longitudinal population studies, a total of 10,367 at T1, 11,585 at T2, and 12,179 at T3 individuals were included in the analyses. Before the start of the pandemic, 30.1% of the population reported working from home (data only available in USOC). This figure increased at T1 with percentages ranging between 32.9 and 65.5 across studies (Table 1). Percentages after combining working fully or partially from home were between 28.8 (in NCDS) and 64.7 (in BiB) at T2 and between 36.5 (in NCDS) to 64.2 (in GS) at T3. See S5 and S6 Supplementary Files for a detailed overview of descriptive statistics for exposure, covariates, and outcome variables.

**Table 1:** Home working by time point across the 7 studies (weighted data).

	Pre-pandemic		Apr–Jun 2020		Jul–Oct 2020			Nov 2020–Mar 2021		
	N	Proportion working from home (%)	N	Proportion working from home (%)	N	Proportion fully working from home (%)	Proportion partially working from home (%)	N	Proportion fully working from home (%)	Proportion partially working from home (%)
Next Steps—NS			943	65.5	1,988	27.8	16.8	2,301	37.8	14.5
1970 British Cohort Study—BCS			1,873	56.5	2,614	25.7	15.3	2,921	33.3	16.2
1958 National Child Development Study—NCDS			1,169	42.2	1,729	18.5	10.3	1,813	24.1	12.4
English Longitudinal Study of Ageing—ELSA			659	32.9	NA	NA	NA	709	23.1	16.5
Understanding Society—USOC	1,101*	30.1*	3,658	59.3	3,918	23.4	22.0	3,348	36.8	17.6
Generation Scotland—GS			1,695	59.8	1,200	41.3	18.7	1,087	42.9	21.3
Born in Bradford—BiB			370	48.7	136	64.7	NA	NA	NA	NA
Total	1,101		10,367		11,585			12,179		

N.B. For USOC, the descriptive statistics in Table 1 are based on data from a single wave at each time point (T1: May 2020; T2 September 2020; T3: January 2021), thus these samples differ from those used in the main analyses, which are based upon data from multiple waves.

*USOC is the only study with data on pre-pandemic home working. These data are from retrospective questions in the May 2020 wave (i.e., T1).

### Association between home working and social and mental wellbeing

**Fig 1:**
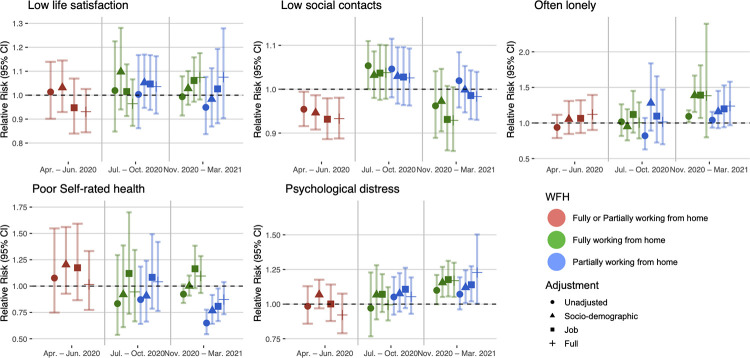
Main model estimates.

Details of study-specific main estimates (RQ1) are presented in S7 Supplementary File with meta-analysed findings presented in Fig 1.

There was no association between full or partial home working and low life satisfaction at T1, T2, or T3.

For social contact, working from home was associated with decreased risk of low social contact (fully adjusted RR: 0.93; 95% confidence interval (CI): 0.89 to 0.98) at T1. At T2, the association was attenuated for both fully and partially working from home, even reversing slightly (e.g., unadjusted RR for full home working: 1.05; 95% CI: 1.00 to 1.10). At T3, fully working from home was again associated with decreased risk of low social contact (fully adjusted RR: 0.93; 95% CI: 0.86 to 1.01), while the association for partial home working was still relatively attenuated (fully adjusted RR: 0.98; 95% CI: 0.93 to 1.04).

Regarding loneliness, there was little evidence for associations between home working and often feeling lonely at T1 and T2. At T3, fully working from home was associated with increased risk of often feeling lonely across the 4 levels of adjustment but associations were imprecisely estimated (fully adjusted RR: 1.38; 95% CI: 0.80 to 2.39). Partially working from home at T3 was similarly associated with often feeling lonely (fully adjusted RR: 1.24; 95% CI: 0.97 to 1.58).

For poor self-rated health, evidence did not support an association with fully working from home at any time point. Partially working from home was associated with reduced risk of poor self-rated health at T3 (RR: 0.81; 95% CI: 0.67 to 0.98), but this was attenuated with full adjustment (RR: 0.87; 95% CI: 0.73 to 1.04).

There was some indication of home working being associated with lower risk for psychological distress at T1 (RR = 0.92, 95% CI = 0.79 to 1.08) and T2 (fully working from home RR = 0.99, 95% CI = 0.88 to 1.11), but CIs overlapped the null. At T3, home working was associated with increased psychological distress for both fully working from home (fully adjusted RR: 1.17; 95% CI: 1.05 to 1.30) and partially working from home (fully adjusted RR: 1.22; 95% CI: 1.00 to 1.48).

### Stratification by sex, age, education, and working time pattern

**Fig 2:**
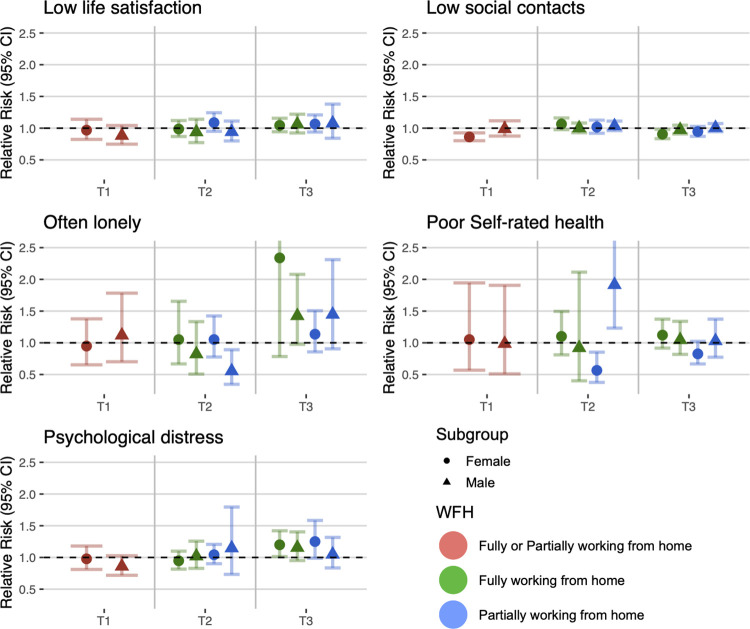
Stratification by sex.

**Fig 3:**
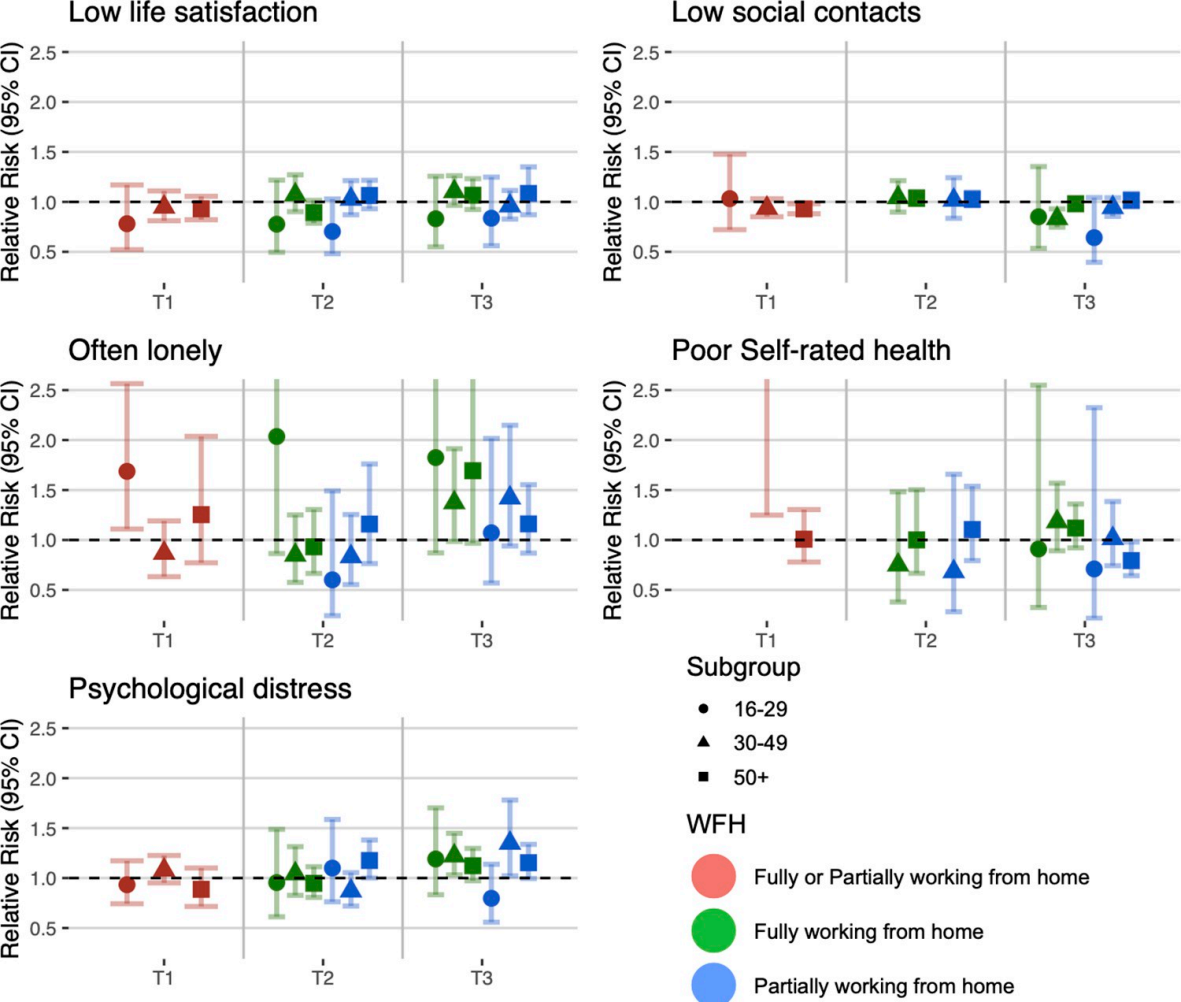
Stratification by age group.

**Fig 4:**
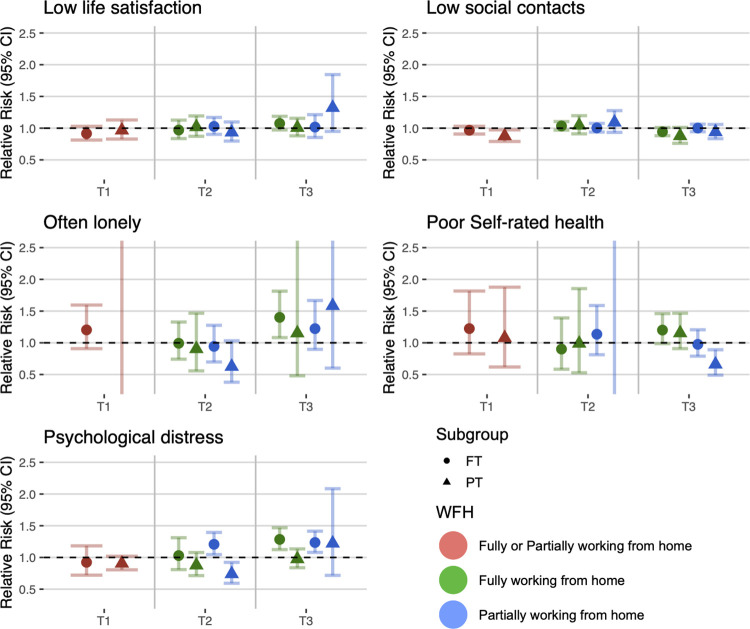
Stratification by full-time and part-time work.

**Fig 5:**
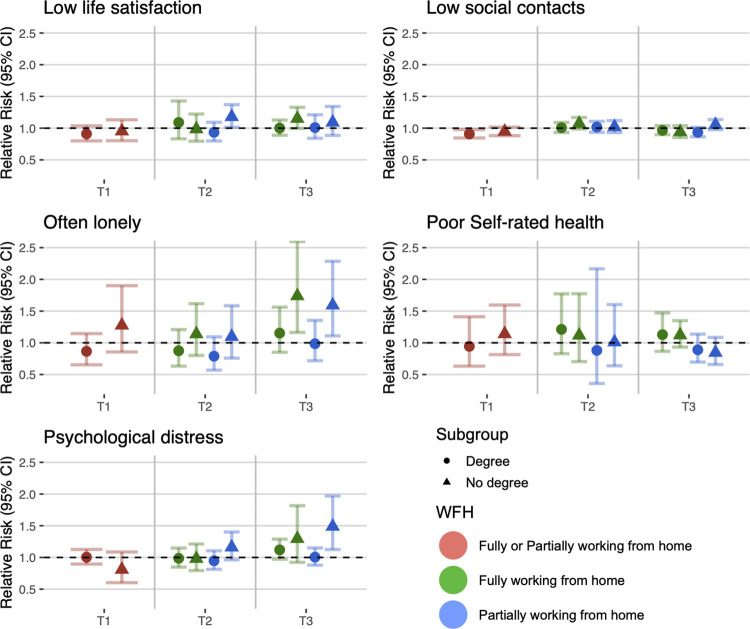
Stratification by highest level of education.

Figs 2–5 show the estimates from the stratified meta-analyses (RQ2; full details of between-group heterogeneity tests are in S8 Supplementary File). The observed associations between home working and social and mental wellbeing measures largely did not differ by sex, age, education, or full versus part-time work.

Looking at sex (Fig 2), associations between home working and reduced risk of low social contact appeared concentrated among females (T1 RR: 0.86; 95% CI: 0.80 to 0.93; T3 fully working from home RR: 0.91; 95% CI: 0.83 to 0.98) rather than males (T1 RR: 0.98; 95% CI: 0.87 to 1.11; T3 fully working from home RR: 0.97; 95% CI: 0.91 to 1.05) although confidence intervals overlapped. At T2, males who were partially working from home had reduced risk of often feeling lonely (RR: 0.55; 95% CI: 0.35 to 0.89) with no clear association among females (RR: 1.05; 95% CI: 0.78 to 1.42). Among those partially working from home, males showed increased risk of poor self-rated health (RR: 1.91; 95% CI: 1.23 to 2.98) in contrast to a reduced risk for females (RR: 0.57; 95% CI: 0.38 to 0.85).

Looking across age-groups (Fig 3), the association between fully working from home and reduced risk of low social contact at T3 was clearer in the 30 to 49 age group (RR: 0.83; 95% CI: 0.75 to 0.93) than in the 50+ age group (RR: 0.98; 95% CI: 0.92 to 1.04). For the 16 to 29 year age group, there was evidence that home working was associated with increased risk of loneliness at T1 (RR: 1.69; 95% CI: 1.11 to 2.56). At T1, working from home was clearly associated with increased risk of poor self-rated health for respondents aged 30 to 49 (RR: 2.74; 95% CI: 1.25 to 5.99), but not for those aged 50+ (RR: 1.01; 95% CI: 0.78 to 1.30), while no estimate was available for those aged 16 to 29 years.

Looking at part-time versus full-time work (Fig 4), partial home working was associated with increased risk of psychological distress for those working full time at T2 (RR: 1.21; 95% CI: 1.05 to 1.39) but decreased risk for those working part time (RR: 0.74; 95% CI: 0.59 to 0.92). Working fully from home was associated with increased psychological distress for those in full-time work at T3 (RR: 1.28; 95% CI: 1.12 to 1.47), but not for those in part-time work (RR: 0.98; 95% CI: 0.84 to 1.13).

Finally, looking at education (Fig 5), there was increased risk of low life satisfaction among respondents with no degree for partial home working at T2 (RR: 1.18; 95% CI: 1.01 to 1.37) and for full home working at T3 (RR: 1.15; 95% CI: 1.00 to 1.33) but not among participants with a degree. Furthermore, at T3 for both loneliness and psychological distress, there was a pattern whereby home working was more clearly associated with poor wellbeing among those with no degree (e.g., partial home working RR for distress: 1.49; 95% CI: 1.12 to 1.97) than those with a degree (RR: 1.01; 95% CI: 0.88 to 1.15).

Heterogeneity statistics for each meta-analyses are presented in S7 Supplementary File. They typically suggest a low level of between-study variability (as evidenced by small tau^2 values) but for some meta-analyses, the between-study variability was somewhat greater.

### Sensitivity analyses

S9 Supplementary File details re-analysis of the USOC data for the main model, using observed pre-pandemic home working (rather than the imputed values), showing consistent results. Additional analyses were also made excluding BiB and GS due to lack of consistency in the control and exposure variables with similar results observed (see S10 Supplementary File) except for the association between loneliness and fully working from home that became significant at 95% at T3 (fully adjusted RR: 1.52; 95% CI: 1.12 to 2.18) but the point estimate of the RR did not change markedly (RR: 1.38; 95% CI: 0.80 to 2.39).

## Discussion

Analysing data from 7 UK longitudinal population studies with adjustment for a range of confounding factors, we found little supporting evidence for an association between home working and lower social and mental wellbeing during the first UK lockdown. Although there was some evidence that home working increased social contact during this period.

Little evidence of associations between home working and social and mental wellbeing were found at T2, when restrictions were eased during summer 2020. Although, the study suggests that some population subgroups may have been more affected, as partial home working was associated with increased risk of psychological distress for those aged 50 or over and for those working full time. We also found an increased risk of poor self-rated health for males and increased risk of low life satisfaction for those with less than degree-level education.

When lockdown measures were re-introduced in the UK (winter 2020 to 2021), there was evidence that home working (full or partial) was associated with increased risk of loneliness and psychological distress, especially for those aged 30 to 49 years, those with no educational degree, and those in full-time work. Together, this suggests that the associations with home working during the pandemic were complex and varied across time points and population subgroups.

Workers have experienced tremendous disruptions due to the pandemic [25,26], with many losing their job, being furloughed [27], experiencing changes in working hours [28], or shifting to working from home [29,30]. The impact of employment disruptions on mental and social wellbeing has been investigated [27], but little was known about the role of home working. The clearest pattern of results emerged at T3, when lockdown measures were reintroduced, when those working either partially or fully from home showed an increased risk for psychological distress and loneliness. At that time, the UK population was almost 1 year into the COVID-19 pandemic, so the finding could represent people experiencing “lockdown fatigue” in relation to home working. As the pandemic progressed, people were also increasingly returning to their workplace, so another explanation of these findings could be that those with poor mental wellbeing were more likely to maintain home working. Results appeared to be stronger in the age 30 to 49 bracket, for those without a degree, and for those working full time. These groups may have faced additional pressures on their time, due to childcare and home-schooling responsibilities. This finding shows that the relation between home working and social and mental wellbeing could be particularly sensitive to the overall pandemic context as well as the kind of work arrangement that is implemented. We also found a small decreased risk for low social contact among home workers during lockdowns. We speculate this could have been driven by more frequent social contact (e.g., via phone calls and messaging) that could have mitigated increased loneliness and poor mental health.

Pre-pandemic evidence suggests that home working is associated with multiple benefits, including greater employee productivity, work satisfaction, better perceived work–life balance, and reduced sick leave [31]. However, potential negative effects of home working have also been reported, such as increased levels of stress and social isolation [16]. Recent reviews of the pre-pandemic literature confirmed a mixed evidence-base and highlighted existing limitations including a lack of multidimensional approaches, whereby studies consider multiple aspects of physical and psychosocial health, and sparsity of longitudinal studies [13,16,32]. Nevertheless, it is unclear to what extent past evidence translates to home working experiences during the COVID-19 pandemic, when home working was rapidly enforced for many and occurred in combination with other public health mitigation measures (e.g., social distancing or school closures).

Several longitudinal studies have investigated changes in workers’ mental health and wellbeing from before to during the first UK lockdown. Pelly and colleagues [33] identified a general pattern of improved wellbeing in workers during the first full UK lockdown (May to July 2020). Studies have primarily suggested positive effects of working from home [10–12], with a few exceptions [14], for example, Giovanis and Ozdamar [15] found that home working during the pandemic was associated with poorer mental health compared to those working at employer’s premises, but only for those working fully from home. More recent studies have suggested that impacts of home working on mental and social wellbeing during the pandemic have been especially strong among women and mothers [34,35] and keyworkers [36]. Further studies have also shown that effects may have changed throughout the pandemic but evidence for this has been mixed [37].

Our analyses add to this literature using data from 7 large UK population studies, with rich pre-pandemic information and multiple waves of data collection. Our pooled analyses have been extensively developed during the pandemic [27,38–40] and offer considerable statistical power to examine whether associations with home working differed over time or between population subgroups. Examining multiple indicators of mental health and social wellbeing, we provide robust evidence on the associations between pandemic home working and specific aspects of social and mental wellbeing. Our findings confirm that associations differ between full and partial modes of home working, between population subgroups, and over time.

Alongside the abovementioned strengths, we note limitations. Firstly, despite adjustment for confounders, unobserved confounding could still have affected our estimates, as with most observational studies. Pre-pandemic home working was unobserved in most studies, and therefore, propensities for home working were imputed using the 2019 to 2020 Annual Population Survey. Although, sensitivity analyses conducted with data from USOC (which did collect information on pre-pandemic home working) produced similar results to those produced using imputed home working propensities.

Secondly, despite adjustment for pre-pandemic wellbeing, it is possible that changes in wellbeing after measurement or during the pandemic influenced the likelihood of home working, so findings may represent reverse causality. Furthermore, while we applied study-specific weights to account for sampling design and differential nonresponse, residual selection bias may remain.

Thirdly, a limitation concerns the definition of home working itself. There are several ways to categorise the default place of work [41]. The International Labour Organisation distinguishes 4 concepts that overlap. First, “remote work” is described as a situation where the work is fully or partially carried out at an alternative worksite other than the default place of work. Second, “telework” is similar to remote work except that it involves the use on the worker’s personal electronic devices. Third, “work at home” is described a situation in which the work takes place fully or partially at home. Fourth, “home-based work” described a situation where work is usually carried out at home, regardless of whether this is the default place of work. These 4 concepts overlap and raise questions on how statistical analyses capture home working and how to define home working during the pandemic as many countries, including the UK, have imposed, for a defined lapse of time, home working for most workers. They also show that home working is a matter of intensity with some workers working fully from home and some others working partially within their workplace. Most of the datasets used in this study did not include information on home working hours with only a distinction between full and partial home working. Minor differences were found between full and partial home working, but future studies should investigate home working intensity more accurately. Similarly, further studies on the relationship between home working and mental health should focus on whether home working (fully or partially) is voluntary and how it is implemented within companies, focusing both on the employees’ willingness to work from home and on the role of collective negotiation in implementing these schemes [42].

Fourthly, information on home working at T1 was collected on a binary basis (working from home or not) in most studies and we could not distinguish full from partial home working as we have done for T2 and T3. Given that T1 corresponds to the first COVID-19 lockdown, it can be assumed that a large part of the workforce was fully working from home but this lack of information is a data limitation.

Finally, although there was a low level of between-study variability for most meta-analyses, the between-study variability was somewhat greater for some. Reasons for this heterogeneity could be explored in future work.

With many workers in the UK now maintaining home working arrangements in some form, this study’s findings, from the summer 2020 period when restrictions had eased, can be particularly illustrative for informing working from home post-pandemic. We found no overall association with poor social and mental wellbeing during this period, indicating that home working arrangements might continue without detrimental impacts to population mental health. These estimates flow from a period when pandemic-related restrictions were eased, although may not be directly comparable to post-pandemic times. Furthermore, as home working arrangements continue, further monitoring of mental health is essential, especially looking at differences by sex, age, education, and working time.

## Conclusions

Home working became more prevalent during the pandemic, but as many continue in these working patterns, it is important to understand potential public health impacts. Our findings suggest limited adverse impacts of increased home working on social and mental wellbeing. We only found evidence of home working being associated with increased risk for loneliness and psychological distress during a period when lockdown measures had been re-introduced, during the winter of 2020 to 2021. There was no overall association with such outcomes in the preceding period when restrictions had been eased. Although, there was some indication during the period of eased restrictions that partial home working may have been associated with increased risk for poor outcomes in certain population subgroups (males, full-time workers, those with lower education, and aged 50+). Continued investigation and monitoring is advised to ensure that home working arrangements do not lead to widening inequalities in social and mental wellbeing.

## Supporting information

S1 STROBE Checklist STROBE checklist. (DOCX)

S1 Supplementary File Study description. (DOCX)

S2 Supplementary File Data availability by time point. (DOCX)

S3 Supplementary File Variables description across the 7 studies. (DOCX)

S4 Supplementary File Pre-pandemic home working probabilities by occupation, age-group, and sex. (DOCX)

S5 Supplementary File Descriptive statistics (exposure and covariates). (DOCX)

S6 Supplementary File Outcome descriptive by exposure. (DOCX)

S7 Supplementary File Pooled analyses for the main results (Forest plots). (DOCX)

S8 Supplementary File Subgroup analyses for stratified results. (DOCX)

S9 Supplementary File Sensitivity analyses of self-reported vs. imputed pre-pandemic home working (USOC). (DOCX)

S10 Supplementary File Main estimates (excluding BiB and GS). (DOCX)

## Abbreviations

CI: confidence interval
COVID-19: Coronavirus Disease 2019
ELSA: English Longitudinal Study of Ageing
NCDS: National Child Development Study
NHS: National Health Service
NS: Next Steps
RQ: research question
RR: risk ratio
SOC: Standard Occupational Classification
USOC: UK Household Longitudinal Study
