# Supplementary material for: Home working and social and mental wellbeing at different stages of the COVID-19 pandemic in the UK: Evidence from 7 longitudinal population surveys
Source: PLoS Med. 2023 Apr 27;20(4):e1004214. doi: 10.1371/journal.pmed.1004214 (PMC10138202; doi:10.1371/journal.pmed.1004214)
Supplement: S7 Supplementary File — (DOCX) [file pmed.1004214.s008.docx]

**Supplementary file S7. Pooled analyses for the main results (Forest plots)**

[No adjustment 3](#_Toc108189354)

[Low life satisfaction 3](#_Toc108189355)

[Often lonely 7](#_Toc108189356)

[Psychological distress 11](#_Toc108189357)

[Poor self-rated health 15](#_Toc108189358)

[Low social contact 18](#_Toc108189359)

[Socio-demographic adjustment 21](#_Toc108189360)

[Low life satisfaction 21](#_Toc108189361)

[Often lonely 25](#_Toc108189362)

[Psychological distress 29](#_Toc108189363)

[Poor self-rated health 33](#_Toc108189364)

[Low social contact 36](#_Toc108189365)

[Job adjustment 39](#_Toc108189366)

[Low life satisfaction 39](#_Toc108189367)

[Often lonely 43](#_Toc108189368)

[Psychological distress 47](#_Toc108189369)

[Poor self-rated health 51](#_Toc108189370)

[social contact 53](#_Toc108189371)

[Full adjustment 57](#_Toc108189372)

[Low life satisfaction 57](#_Toc108189373)

[Often lonely 61](#_Toc108189374)

[Psychological distress 64](#_Toc108189375)

[Poor self-rated health 68](#_Toc108189376)

[Low social contact 71](#_Toc108189377)

# No adjustment

## Low life satisfaction


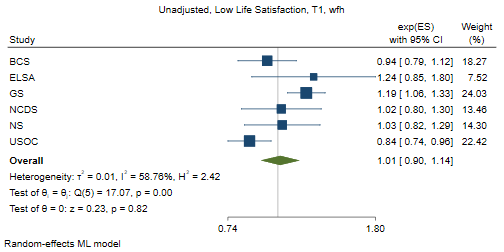

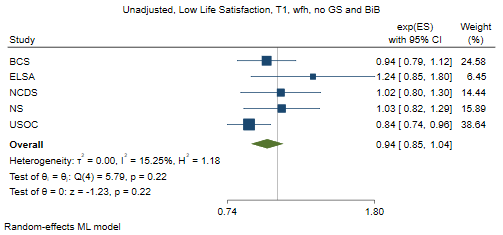

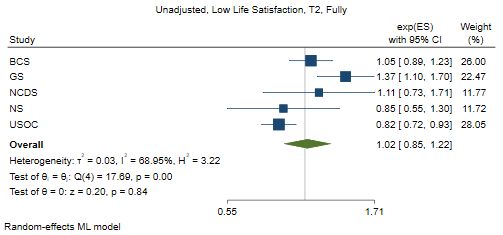

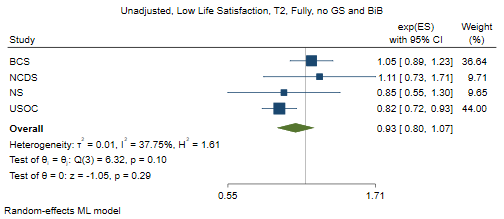

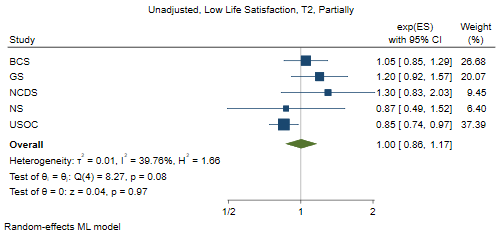

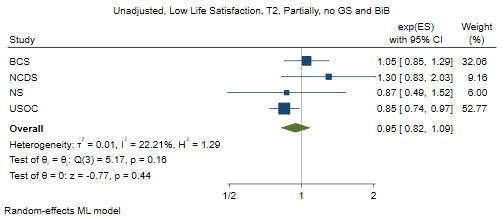

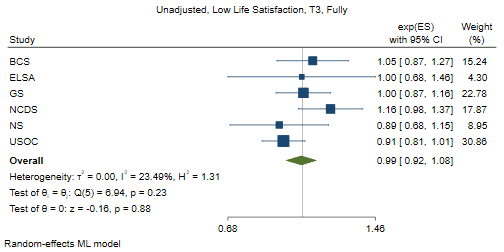

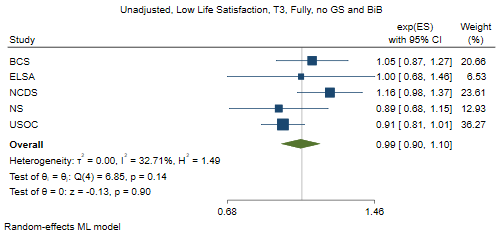

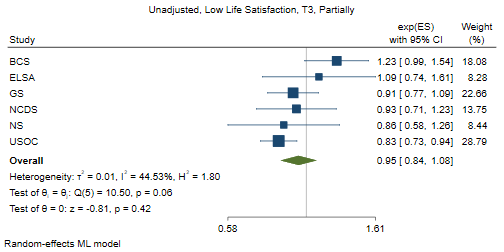

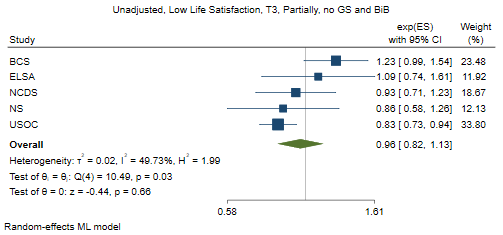


## Often lonely


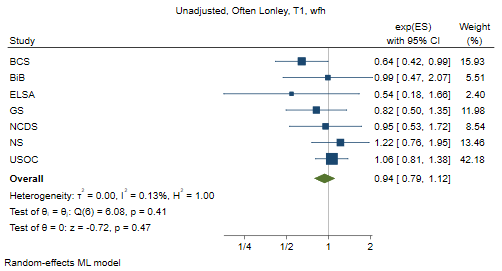

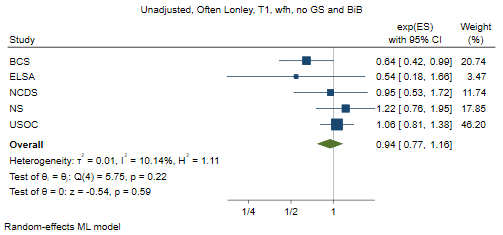

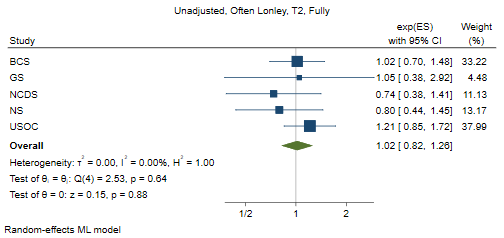

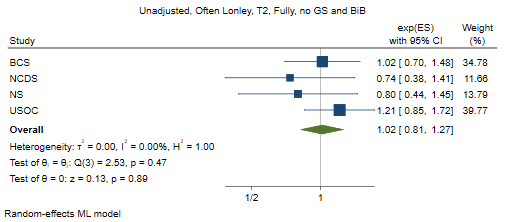

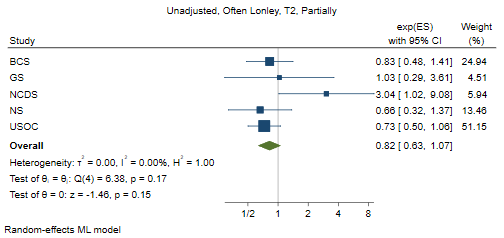

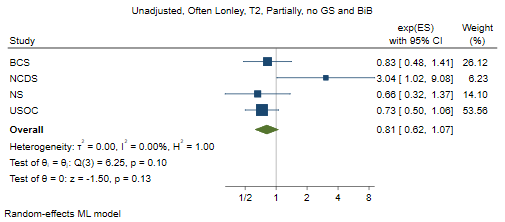

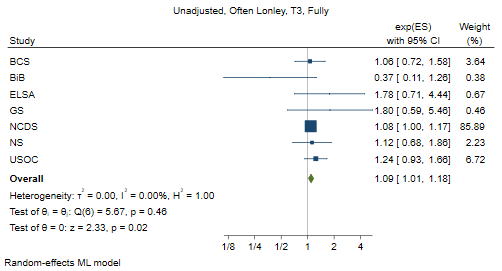

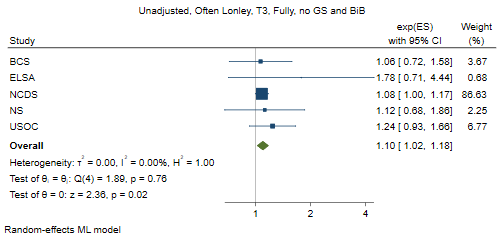

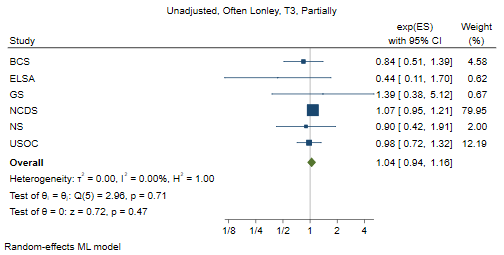

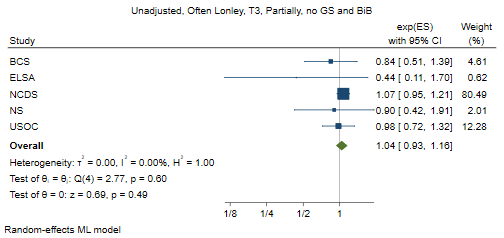


## Psychological distress


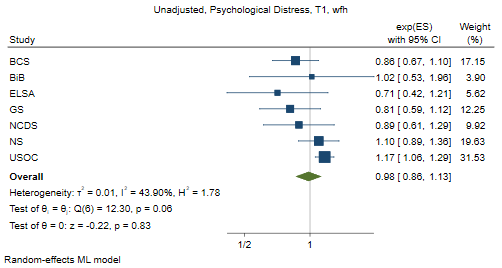

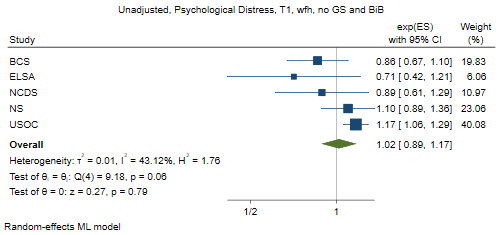

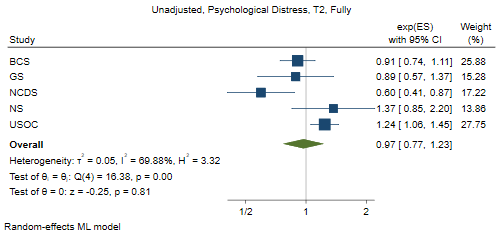

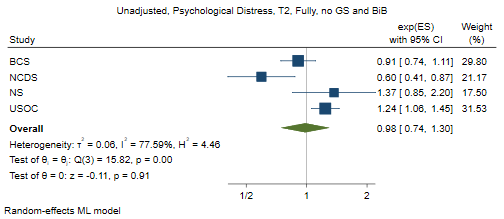

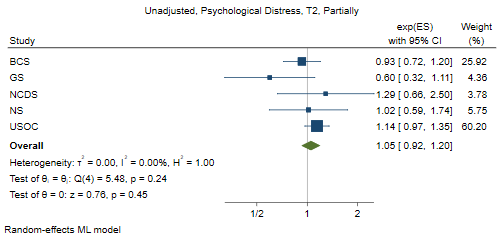

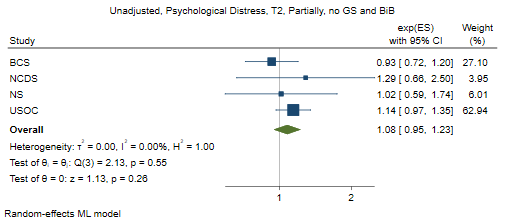

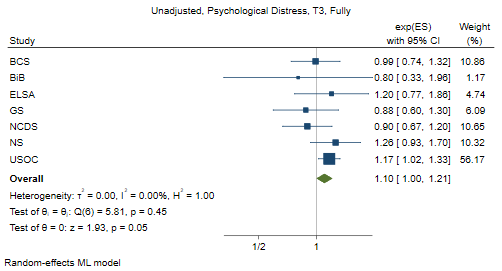

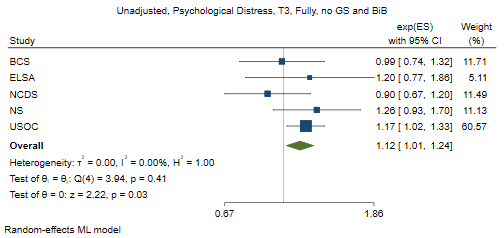

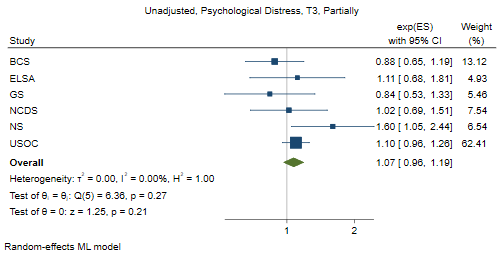

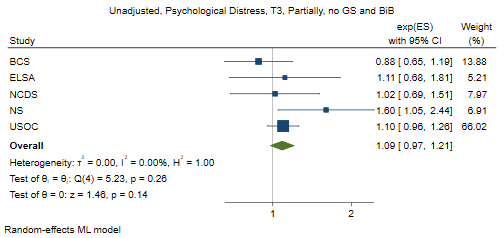


## Poor self-rated health


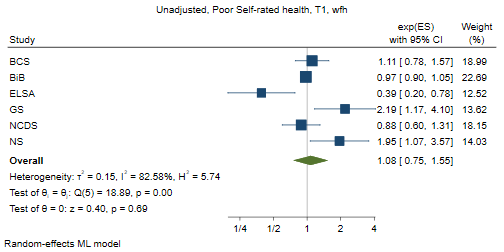

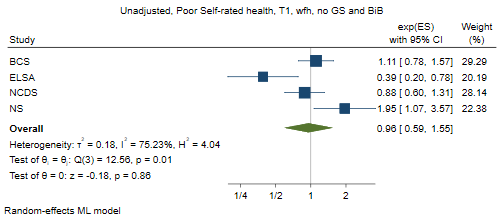

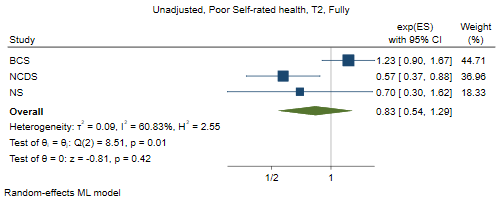

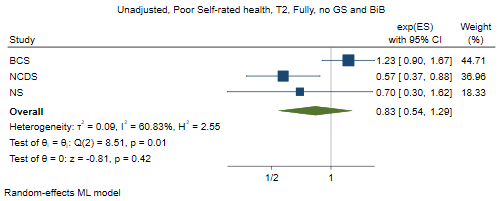

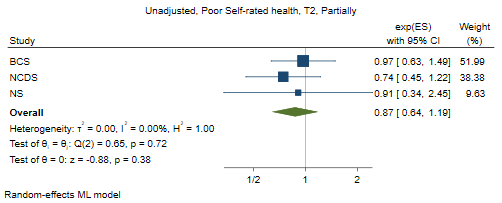

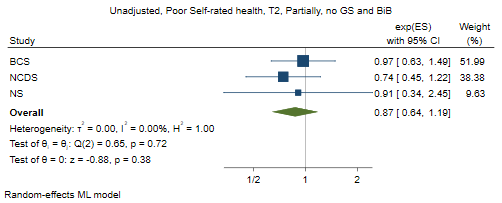

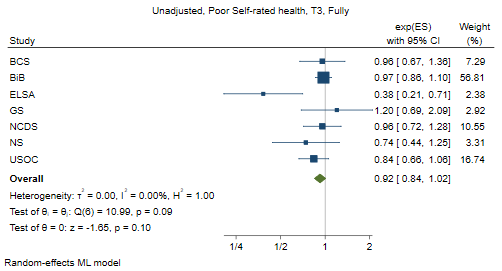

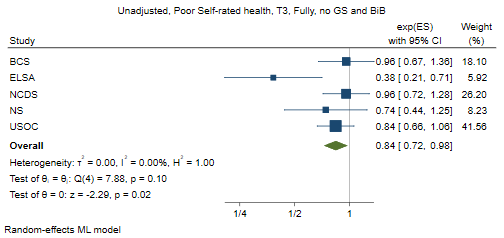

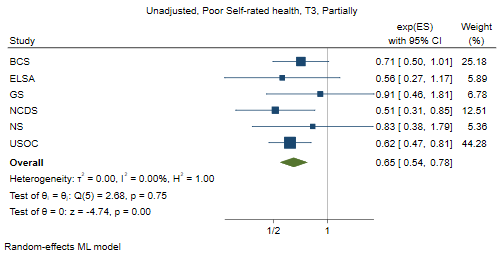

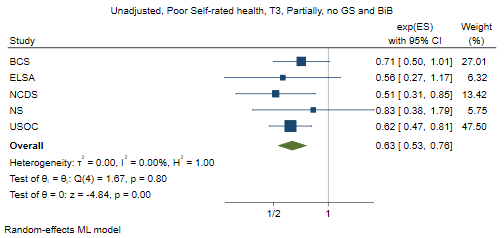


## Low social contact


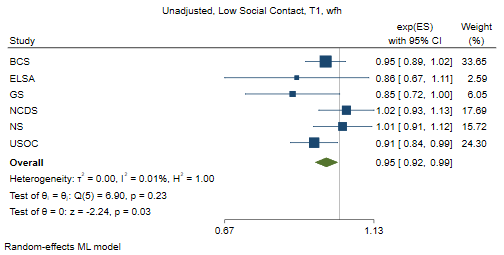

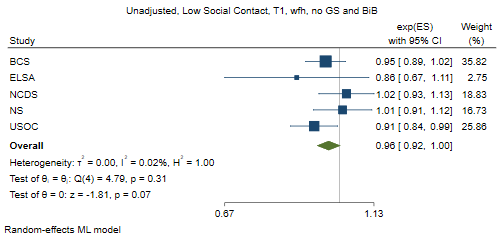

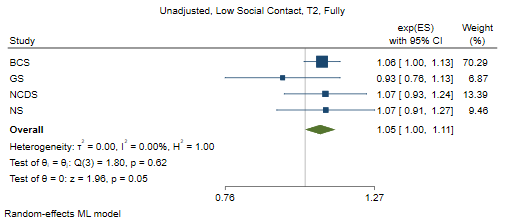

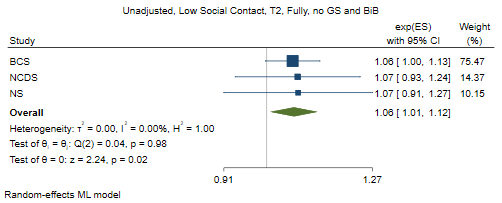

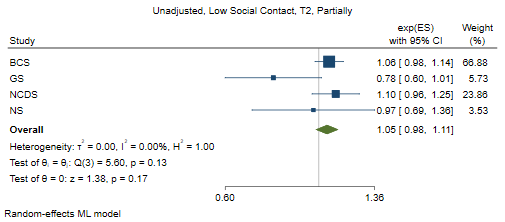

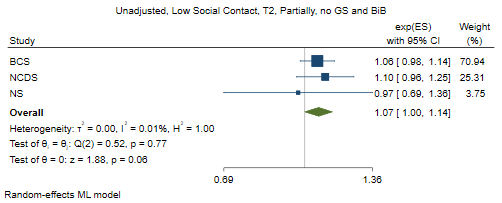

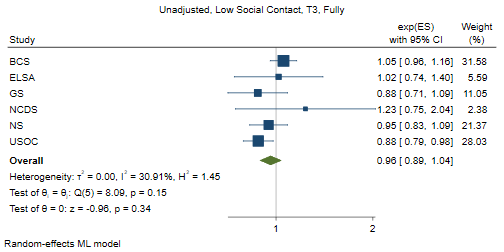

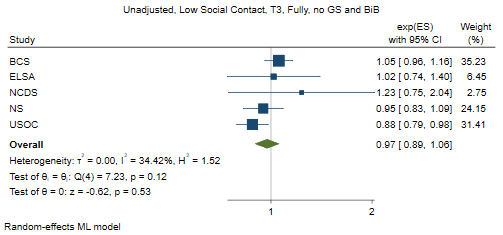

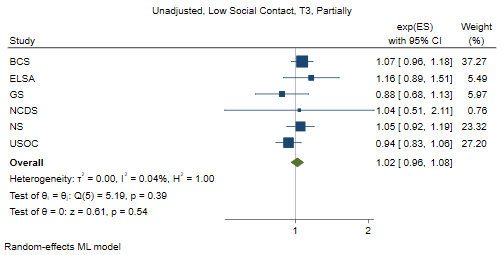

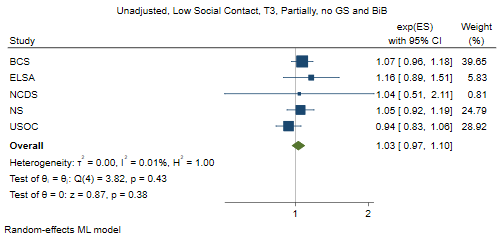


# Socio-demographic adjustment

## Low life satisfaction


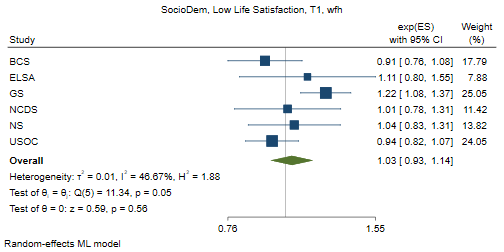

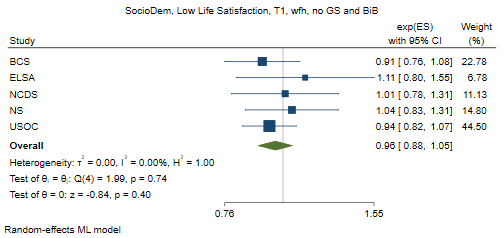

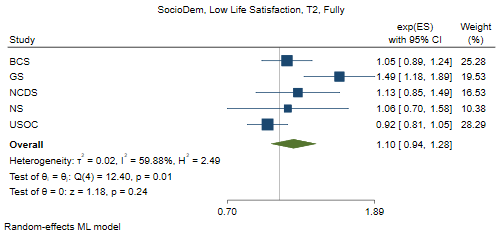

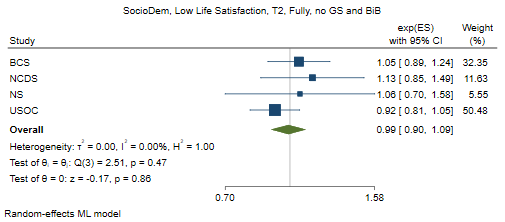

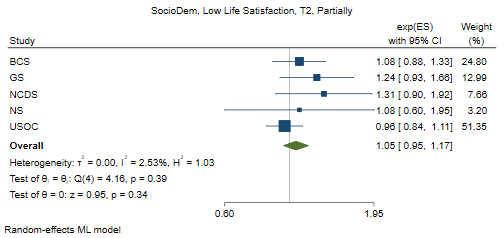

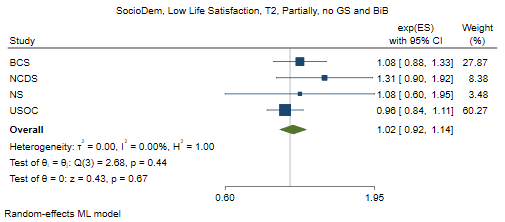

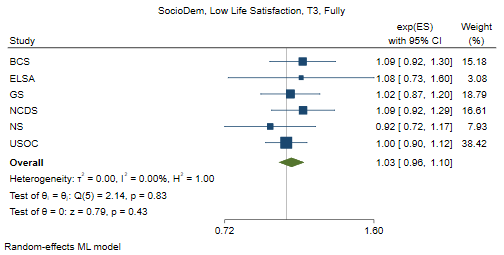

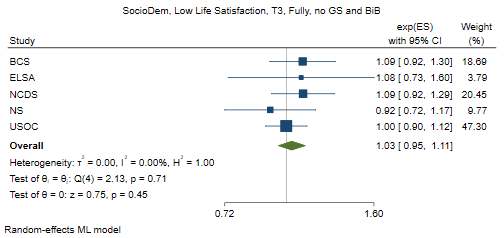

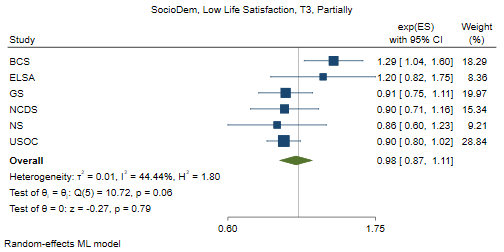

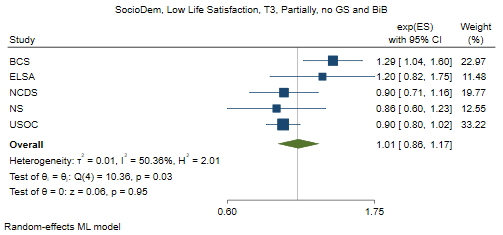


## Often lonely


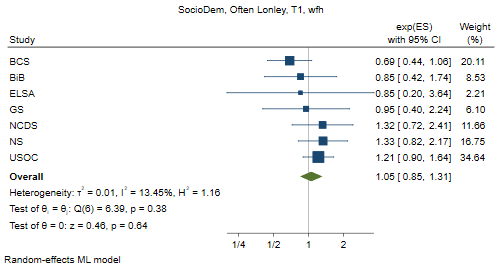

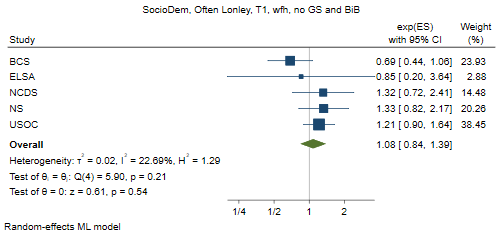

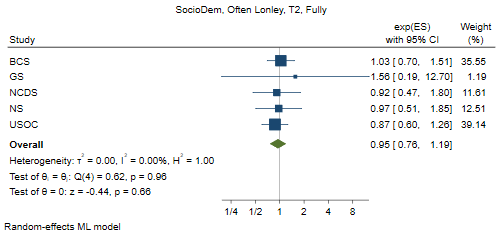

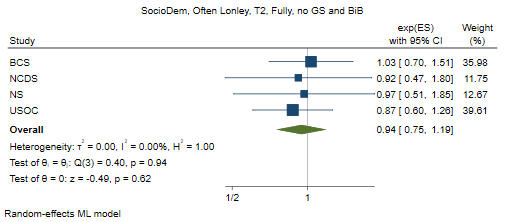

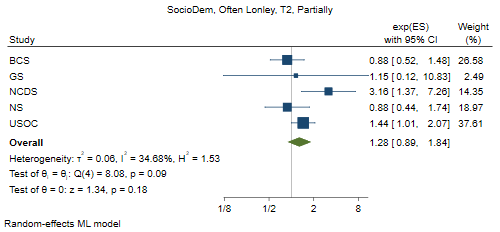

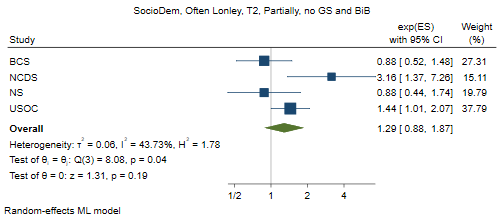

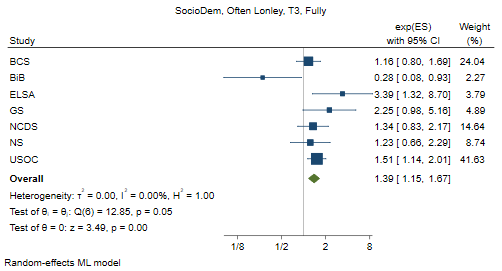

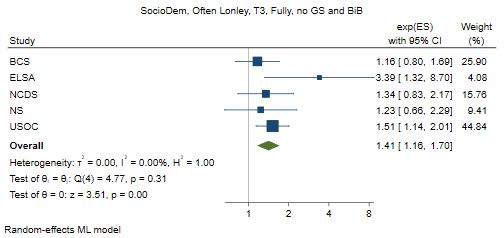

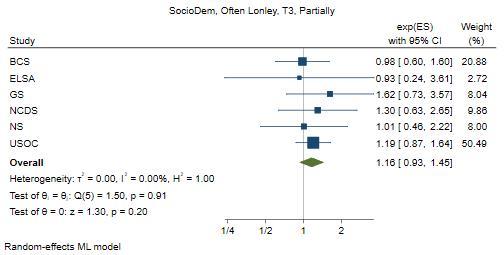

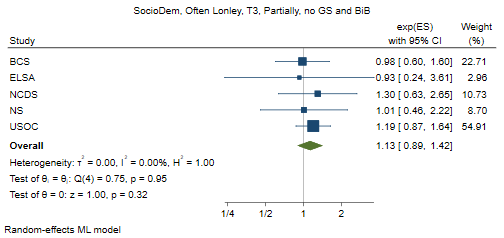


## Psychological distress


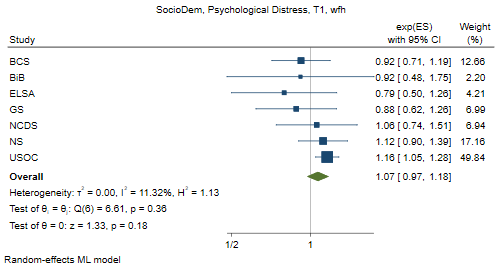

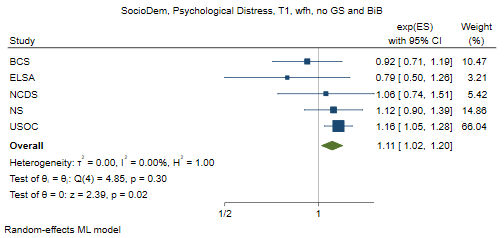

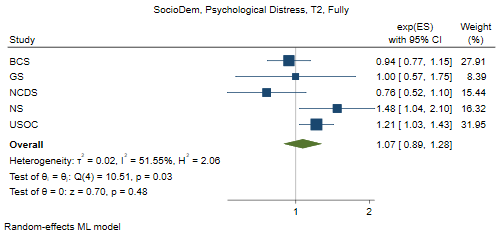

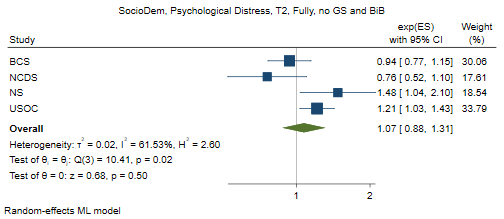

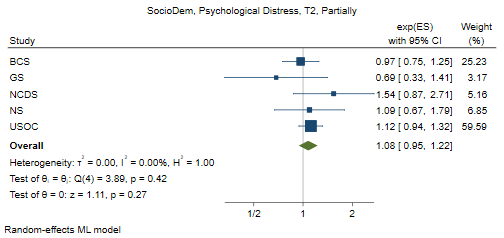

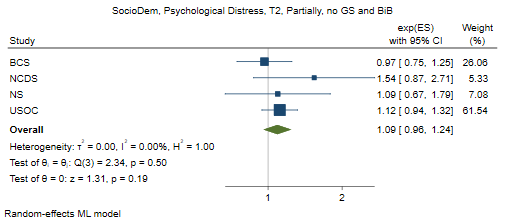

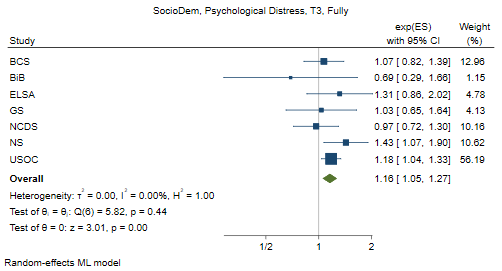

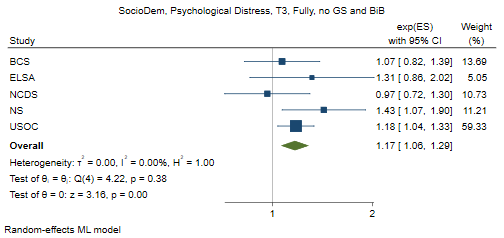

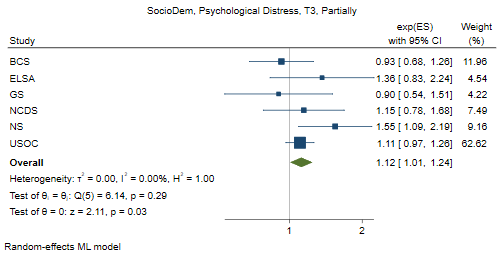

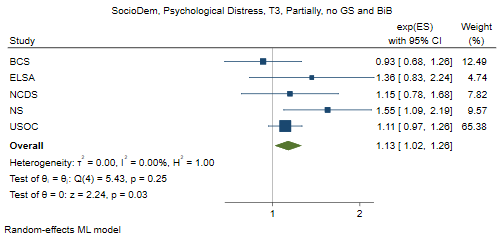


## Poor self-rated health


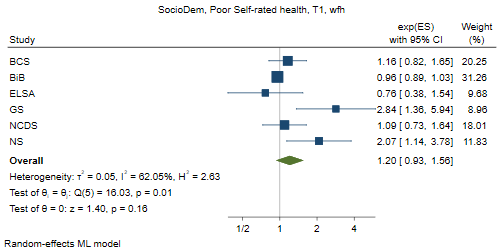

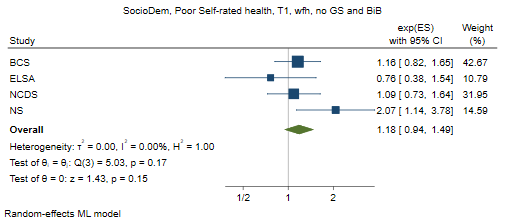

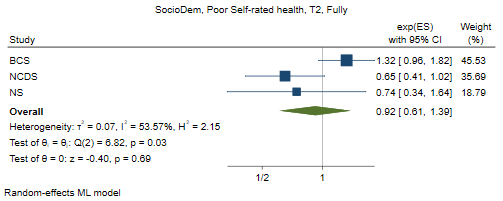

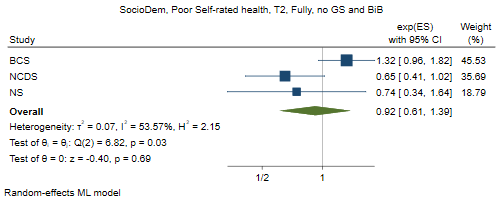

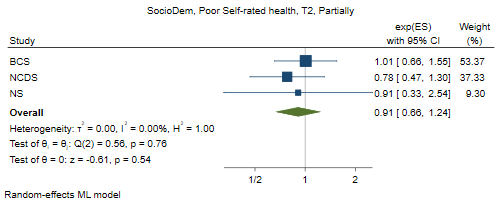

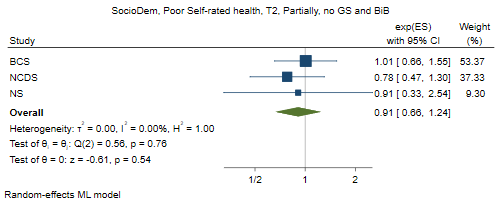

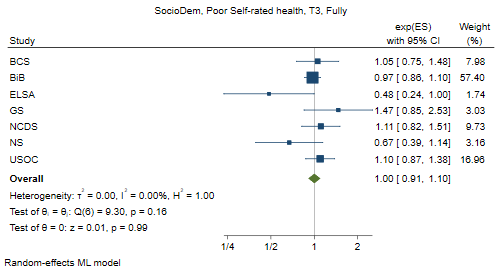

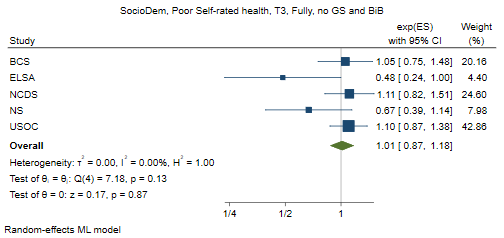

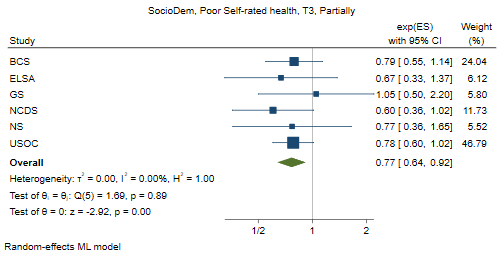

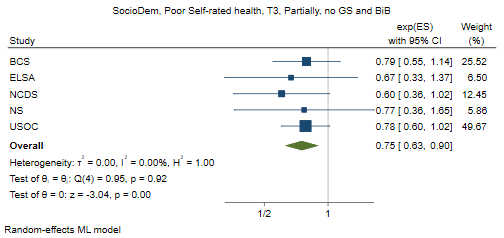


## Low social contact


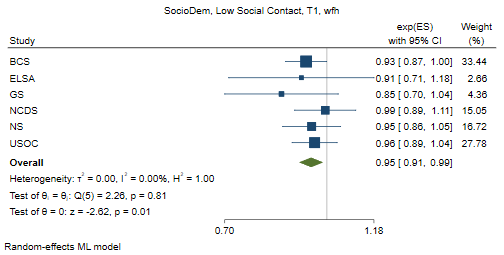

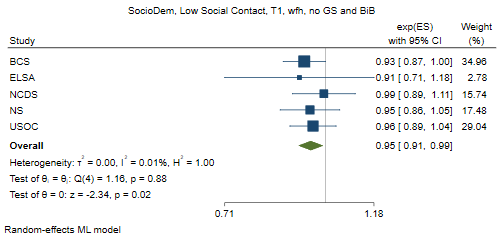

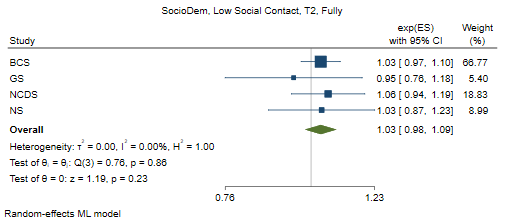

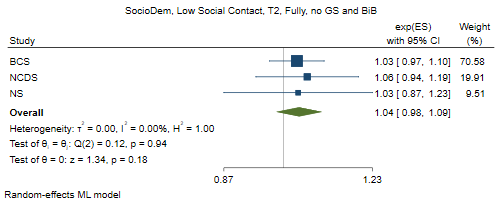

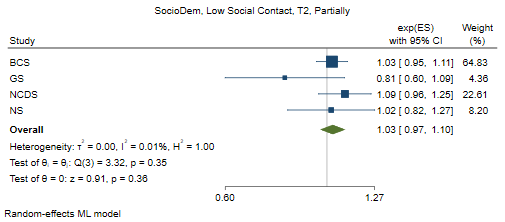

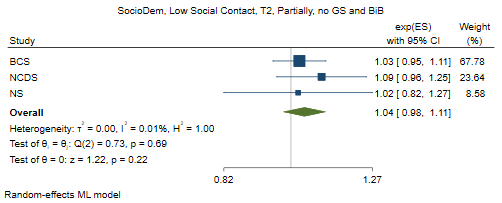

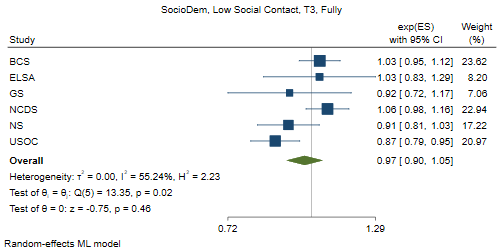

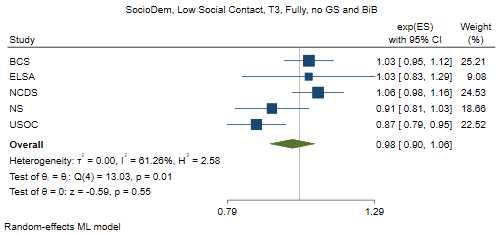

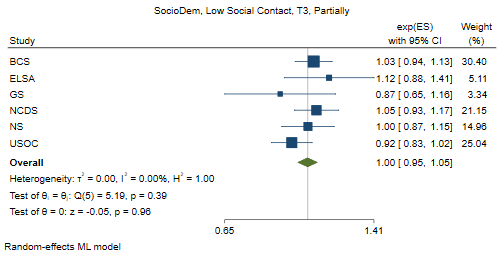

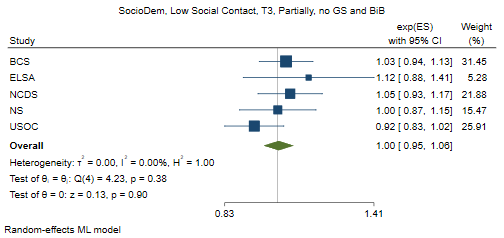


# Job adjustment

## Low life satisfaction

## Often lonely

## Psychological distress

## Poor self-rated health

w

## social contact

# Full adjustment

## Low life satisfaction

## Often lonely

## Psychological distress

## Poor self-rated health

## Low social contact
