# Supplementary material for: Home working and social and mental wellbeing at different stages of the COVID-19 pandemic in the UK: Evidence from 7 longitudinal population surveys
Source: PLoS Med. 2023 Apr 27;20(4):e1004214. doi: 10.1371/journal.pmed.1004214 (PMC10138202; doi:10.1371/journal.pmed.1004214)
Supplement: S10 Supplementary File — (DOCX) [file pmed.1004214.s011.docx]

**Supplementary file S10. Main estimates (excluding BiB and GS)**

**Main model estimates excluding BiB and GS, sensitivity analysis**
